# The influence of subventricular zone involvement in extent of resection and tumor growth pattern of glioblastoma

**DOI:** 10.1515/iss-2020-0011

**Published:** 2020-08-27

**Authors:** Yahya Ahmadipour, Julie-Inga Krings, Laurèl Rauschenbach, Oliver Gembruch, Mehdi Chihi, Marvin Darkwah Oppong, Daniela Pierscianek, Ramazan Jabbarli, Ulrich Sure, Nicolai El Hindy

**Affiliations:** Department of Neurosurgery, University Hospital Essen, Essen, Germany; German Cancer Consortium, Partner Site University Hospital Essen, Essen, Germany

**Keywords:** extent of resection, glioblastoma, multifocality, subventricular zone

## Abstract

**Objectives:**

*Isocitrate dehydrogenase* (*IDH1/2*) mutations and *O6-alkylguanine DNA methyltransferase* (*MGMT*) promoter methylations are acknowledged survival predictors in patients with glioblastoma (GB). Moreover, tumor growth patterns like multifocality and subventricular zone (SVZ) involvement seem to be associated with poorer outcomes. Here, we wanted to evaluate the influence of the SVZ involvement and the multifocal tumor growth on the extent of surgical resection and its correlation with overall survival (OS) and molecular characteristics of patients with GB.

**Methods:**

Adult patients with primary GB who underwent surgery at our department between 2012 and 2014 were included. Preoperative magnetic resonance imaging findings were analyzed with regard to tumor location, presence of multifocality and SVZ involvement. The extent of surgical resection as well as clinical and molecular parameters was collected from electronic patient records. Univariate and multivariate analyses were performed.

**Results:**

Two hundred eight patients were retrospectively analyzed, comprising 90 (43.3%) female individuals with a mean age of 62.9 (±12.26) years and OS of 10.2 months (±8.9). Unifocal tumor location was a predictor for better OS with a mean of 11.4 (±9.4) months (vs. 8.0 [±7.4] months, p=0.008)*.* Affection of the SVZ was also associated with lower surgical resection rates (p<0.001). SVZ involvement revealed with 7.8 (±7.0) months a significant worse OS [vs. 13.9 (±10.1) months, p<0.001]*.* All six *IDH1/2* wildtype tumors showed an unifocal location (p=0.066). *MGMT* promoter methylation was not associated with multifocal tumor growth (p=0.649) or SVZ involvement (p=0.348). Multivariate analysis confirmed independent association between the SVZ involvement and OS (p=0.001).

**Conclusion:**

The involvement of the SVZ appears to have an influence on a lower resection rate of GB. This negative impact of SVZ on GB outcome might be related to lesser extent of resection, higher rates of multifocality and greater surgical morbidity but not inevitably to *IDH1/2* mutation and *MGMT promoter* methylation status.

## Introduction

Glioblastoma (GB) is the most common primary tumor of the central nervous system [1]. Currently, clinical management comprises surgical, chemotherapeutic and radiotherapeutic treatment approaches [2]. Despite tremendous research efforts, early tumor progression and tumor recurrence is a frequent event of GB, whereas the improvement of quality of life is one of the main aims for treatment. However, interindividual patient survival is heterogeneous, with some patients surviving for years [3]. Characteristics like age, clinical performance status, tumor location and molecular tumor markers have been identified as potential prognostic factors in patients with these tumors. Today, *O6-alkylguanine DNA methyltransferase* (*MGMT*) promoter hypermethylation and *isocitrate dehydrogenase* (*IDH1/2*) mutations are important predictors of treatment response and patient survival, allowing a subclassification of patients with malignant gliomas [4], [5], [6].

Numerous preclinical and clinical reports suggest that only a subset of cancer cells within a tumor might be responsible for treatment failure [7], [8]. These cells exhibit stem cell–like properties and are multipotent, proliferative, invasive and resistant to antiglioma treatment. Thus, brain areas with neuronal stem cell activity have early been associated with brain cancer development [9], [10], [11], [12]. The subventricular zone (SVZ) is a brain area close to the lateral ventricles and besides the subgranular zone, which is the most important source of neurogenesis throughout adult life [13]. Preclinical studies demonstrate that neuronal stem cells of the SVZ are able to transform into GB [9], [10], [11], [12]. Moreover, studies have shown a tendency for GB involving the SVZ to poorer outcome with early recurrence and multifocal tumor growth pattern [14], [15], [16], [17]. Finally, there is growing evidence that the SVZ might be more susceptible to tumorigenesis than other brain areas. It is still discussed controversially whether neuronal stem cells are the cell of origin of malignant gliomas [18].

Based on a retrospective single-center cohort with consecutive GB cases, we aimed to analyze the influence of the SVZ involvement and other radiographic tumor growth patterns on overall survival (OS) of patients with GB, particularly with regard to their molecular characteristics.

## Patients and methods

All adult patients (≥18 years) with newly diagnosed GB treated at our department between January 2012 and December 2014 were reviewed retrospectively.

Histology was confirmed in all patients, and neuropathological specimen analysis was performed according to the World Health Organization Classification of Central Nervous System Tumors of 2016 [1]. This study was performed in accordance with the Declaration of Helsinki and was approved by the local ethics committee.

### Pathohistological assessment

Following surgery, patients with a tumor tissue underwent an integrated morphologic and molecular diagnostic analysis at the Institute of Neuropathology at the University Hospital of Essen. O6-methylguanine-DNA methyltransferase promoter methylation was assessed by pyrosequencing. Isocitrate dehydrogenase analysis was performed via immunohistochemistry.

### Magnetic resonance imaging scans

**Figure 1:**
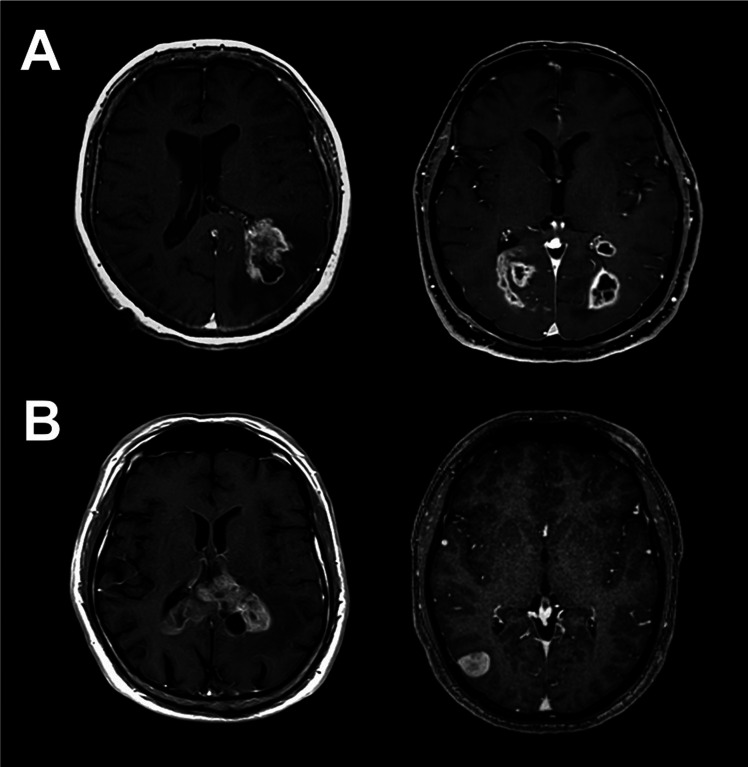
Representative MRI scans of patients with GB. (A) GB with unifocal (left) or multifocal (right) growth pattern. (B) GB with (left) or without (right) SVZ involvement. MRI, magnetic resonance imaging; GB, glioblastoma; SVZ, subventricular zone.

All patients received a preoperative contrast-enhanced magnetic resonance imaging scan to determine tumor extension and SVZ involvement. Tumor location was determined based on contrast-enhanced T1-weighted sequences on axial and coronal images. Per definition, multifocality was defined as the presence of two independent contrast-enhanced lesions in one or both hemispheres. SVZ involvement was assessed if the contrast-enhancing lesion contacted the lateral walls of the lateral ventricle. Examples are shown in Figure 1.

OS after GB surgery was the main endpoint of this study. Moreover, long-term survival was also addressed as two-year survival. Patients’ age was assessed as continuous and dichotomous variables (with dichotomization at the cohort’s mean age). Based on radiological reports, extent of resection (EOR) was evaluated in a categorical manner, where the cases were referred to the following three surgical groups: gross-total resection (GTR, with removal of the contrast-enhancing tumor mass), subtotal resection (STR) and stereotactic biopsy (SB).

SB was conducted using a Leksell Stereotactic System (Elekta, Stockholm, Sweden). In patients with microsurgical resection, a neuronavigation (Brainlab, Munich, Germany) was used, and 5-aminolevulinic acid was given to the patients prior surgery as a standard performance.

Postoperative treatment was planned individually, according to tumor diagnosis, age and clinical performance status. Preoperative and postoperative outcomes were assesses using the Karnofsky Performance Status Scale (KPS) before and up to four weeks after surgery, where KPS ≤70% was defined as poor clinical condition.

The following patient data were collected for further analysis: sex, age, preoperative and postoperative KPS values, EOR, *IDH1/2* mutation and *MGMT promoter methylation status* and OS in months.

The associations of the SVZ involvement with other patients’ characteristics were first analyzed in a univariate manner. Continuous variables were addressed with Pearson’s linear correlation, the Student’s t-test or the Mann-Whitney U tests, as appropriate. Associations between categorical variables were analyzed using the Chi-square test (*χ^2^* test) or Fisher exact test, as appropriate. Kaplan-Meier survival plots for SVZ involvement were also performed. Finally, the predictive value of the SVZ was tested with multivariate linear regression and binary logistic regression analysis for OS and long-term survival endpoints, respectively, adjusting the associations for common confounders (age, EOR, KPS, *IDH1/2* mutation and *MGMT promoter* status). Statistical analyses were performed with SPSS software (IBM,New York, USA, version 24.0). Differences were regarded as significant at p<0.05. Significance values were marked in the tables. 

## Results

### Patient characteristics

A total of consecutive 208 patients with primary GB were included in this study. The mean age was 62.9 (±12.26) years. In our study group, GBs were more common in men (n=118; 56.7%) than in women (n=90; 43.3%). Performed EORs comprised SB (n=46; 22.1%), STR (n=65; 31.3%) or GTR (n=97; 46.6%). The mean KPS before surgery was 78.3% (±13.0); postoperatively, the mean KPS of the cohort declined to 72.9% (±19.0). The *MGMT*-promoter methylation status was investigated in 200 (96.2%) GB individuals whereas 82 (41.0%) were methylated. *IDH 1/* *2* mutational status was available in 195 (93.8%) cases. Here, an *IDH 1/* *2* mutation was determined in 6 patients with GBs (3.1%). The mean OS was 10.2 months (±8.9). Regarding long-term survival, there were 15 (7.2%) cases with two-year survival.

### Multifocality and SVZ involvement

Multifocal location was determined in 77 (37%) patients. Tumor harbored the SVZ in 128 (61.5%) cases. The detailed characteristics of the cohort with special emphasis on SVZ involvement are shown in the Table 1.

**Table 1:** Characteristics different subgroups regarding to SVZ involvement.

	Cases (n)	SVZ+	SVZ−	p-Value
Age years	Mean=62.90, ±12.26	62.96 (±12.91)	62.80 (±11.22)	0.927
OS months	Mean=10.18, ±8.86	7.76 (±7.02)	13.87 (±10.08)	<**0.001**
Two-year survival	n=15	3 (20%)	12 (80%)	**0.001**
KPS preoperative	Mean=78.32%	77.34%	79.88%	0.176
KPS postoperative	Mean=72.93%	70.86%	76.25%	**0.047**
*MGMT* methylated	n=82, 41%	46 (56.1%)	36 (43.9%)	0.380
*IDH1/2* mutated	n=6, 3.1%	3 (50%)	3 (50%)	0.682
Extent of resection (EOR)	GTR (n=97)	43 (44.3%)	54 (55.7%)	<**0.001**
	STR (n=65)	47 (72.3%)	18 (27.7%)	
	SB (n=46)	38 (82.6%)	8 (17.4%)	
Multifocality	Unifocal (n=131)	65 (49.6%)	66 (50.4%)	<**0.001**
	Multifocal (n=77)	63 (81.8%)	14 (18.2%)	

GB, glioblastoma; KPS, Performance Status Scale; OS, overall survival; SVZ, subventricular zone; STR, subtotal resection.

**Figure 2:**
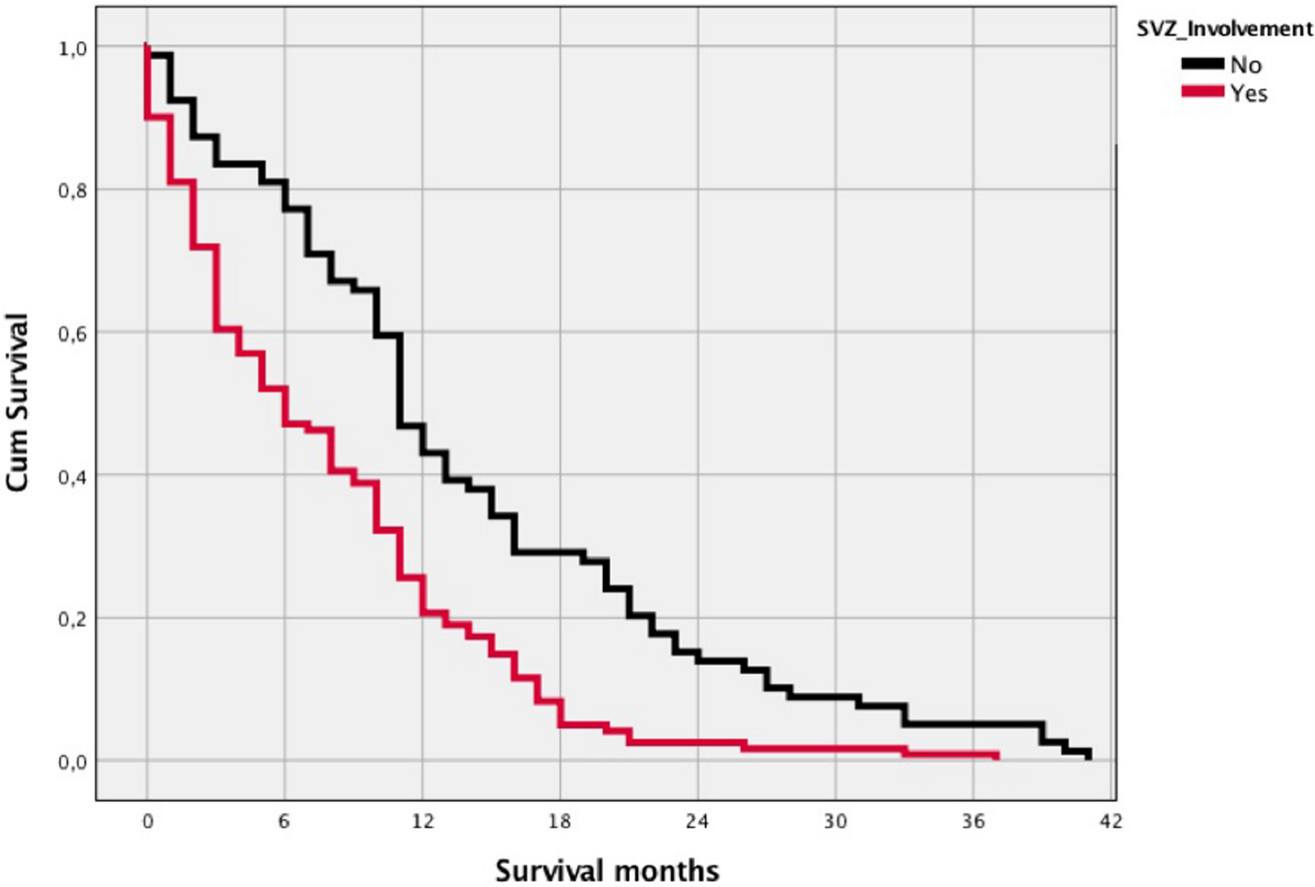
Kaplan-Meier survival curves with OS in months related to SVZ involvement. OS, overall survival; SVZ, subventricular zone.

There was an association between multifocal tumor growth and SVZ involvement in our cohort (p<0.001, see Table 2). In addition, both radiographic parameters were associated with poorer survival of patients with GB: 8.0 (±7.4) vs. 11.4 (±9.4) months for multifocality (p=0.008) and 7.8 (±7.0) vs. 13.9 (±10.1) months for SVZ involvement (p<0.001) (Figure 2). There was a significant association between SVZ involvement and worse two-year survival (p=0.001) which was not observed for mutifocality (p=0.179). *MGMT* promoter status did not show any association with multifocal tumor growth (p=0.649) or SVZ involvement (p=0.380). In turn, all *IDH1/2* mutated tumors showed an unifocal growth pattern (p=0.066) but were equally distributed with regard to SVZ (p=0.682).

**Table 2:** Comparison of SVZ involvement and multifocality in relation to overall survival in months.

GB (n=208)	Survival months	Std. deviation	p-Value
Unifocal	11.4	±9.4	**0.008**
Multifocal	8.0	±7.4	
SVZ uninvolved	13.9	±10.1	**<0.001**
SVZ involved	7.8	±7.0	

GB, glioblastoma; SVZ, subventricular zone.

### Impact of SVZ on surgical outcome

GB cases with SVZ involvement revealed a significant decrease of the KPS after surgery (6.5 vs. 3.6% in cases without SVZ, p=0.047). Affection of the SVZ was also associated with higher rates of SB or STR than GTR (p<0.001)*.* Multifocal tumor characteristic was also correlated with lower EOR (p<0.001, see Table 3). Finally, multivariate analysis confirmed independent association between SVZ involvement and poor OS (p=0.001, see Table 4).

**Table 3:** Influence of multifocality and SVZ involvement in extent of resection.

Extent of resection	Stereotactic biopsy (n=46)	Subtotal resection (n=65)	Gross total resection (n=97)	p-Value
Unifocal (n=131)	20 (15.3%)	37 (28.2%)	74 (56.5%)	**<0.001**
Multifocal (n=77)	26 (33.8%)	28 (36.4%)	23 (29.9%)	
SVZ involved (n=128)	38 (29.7%)	47 (36.7%)	43 (33.6%)	**<0.001**
SVZ uninvolved (n=80)	8 (10%)	18 (22.5%)	54 (67.5%)	

SVZ, subventricular zone.

**Table 4:** Multivariate analysis of predictor of OS.

Parameters	RC	95% CI		p-Value
Age	−0.157	−0.254	−0.060	**0.002**
SVZ involvement	−4.182	−6.535	−1.829	**0.001**
KPS preoperative	0.083	−0.004	0.170	0.062
*MGMT* methylation	2.863	−0.004	0.170	**0.015**
*IDH1/2* mutation	10.679	4.04.3	17.316	**0.002**
EOR	2.213	0.661	3.766	**0.005**

CI, confidence interval; EOR, extent of resection; KPS, Performance Status Scale; RC, regression coefficient; SVZ, subventricular zone.

## Discussion

This study evaluated the relationship between SVZ involvement and other characteristics of patients with GB. The presented study showed higher rate of SVZ involvement in our GB cohort. Tumors within SVZ are characterized with poorer survival, which is most likely related to higher surgical morbidity and lower EORbut not to molecular-genetic characteristics of the patients.

Radiographic contact to the SVZ is a common phenomenon in patients suffering from malignant gliomas [19]. The SVZ represents one major area of neurogenesis and is suspected to be an origin of brain cancer cells with stem cell–like properties [10], [11], [20], [21], [22]. Thus, tumors with SVZ involvement might be more proliferative and invasive. In the last decade, numerous studies have discussed that tumor localization with regard to the SVZ is associated with an increased tumor volume, multifocal tumor growth, early recurrence in patients with GB [14], [15], [16], [17], [23], [24], [25], [26], [27], [28], [29]. This is in line with our data that patients with SVZ involvement were associated with shorter OS.

Consistent with previously published reports [16], [25], [30], there was a high coincidence between multifocality and SVZ involvement in our cohort. The significant incidence of SB in cases with SVZ involvement (38/46, 82.6%) and their association with higher rate of multifocal growth pattern (63/77, 81.8%) in our data could be the main reason for a decision of biopsy with consecutive lower survival rate.

Diagnosis of malignant glioma is currently based on histologic and molecular features [1]. Thus, we also investigated the relationship of *MGMT* promoter methylation and *IDH1/2* mutation status with multifocal tumor growth and tumor contact to the SVZ. *MGMT* promoter status was not associated with location or multifocality, consistent with previously published reports [31], [32]. Former findings from Pińa Batista et al. [33] reported a minor incidence of *IDH1/2* mutated GB with SVZ involvement (n=5, 11.9%) and a major incidence of *IDH1/2* mutated GB distant to the SVZ (n=37, 88.1%). Interestingly, all *IDH1/2* mutated tumors showed an unifocal tumor growth pattern in our cohort. The absence of *IDH 1/2* mutated tumors in multifocal GBs in our cohort could be explanatory one of the reasons for a better prognosis [34]. Hitherto, an equal amount of *IDH 1/2* mutated tumor was observed regarding to SVZ involvement. Here, the number of patients with *IDH 1/2* mutations is probably too low to show a significance.

## Conclusion

Negative impact of SVZ on GB outcome might be related to lower EOR, higher surgical morbidity and higher rates of multifocality but not to IDH1/2 mutation and MGMT promoter methylation status. Further studies are required to elucidate molecular markers relevant for tumor growth patterns and to optimize the treatment strategies of patients with BB and SVZ involvement.
