# Supplementary material for: The influence of subventricular zone involvement in extent of resection and tumor growth pattern of glioblastoma
Source: Innov Surg Sci. 2020 Aug 27;5(3-4):127–32. doi: 10.1515/iss-2020-0011 (PMC8668024; doi:10.1515/iss-2020-0011)
Supplement: Supplementary file 1 [file iss-05-20200011-s001.pdf]

Reviewer Assessment

Yahya Ahmadipour et al.: The influence of subventricular zone involvement on extent of resection and tumor growth pattern of Glioblastoma

Reviewers' Comments to Original Submission

Reviewer 1: anonymous

Date received: 19-May-2020
Reviewer recommendation: **Return to author for minor modifications**
Reviewer overall scoring: **High**

Assessment Form scores: 5 = High/Yes; 3 = Medium/Adequate; 1 = Low

Is the subject area appropriate for the journal	5				
Does the title clearly reflect the paper's content?	5				
Does the abstract clearly reflect the paper's content		4			
Do the keywords clearly reflect the paper's content?		4			
Does the introduction present the problem clearly?		4			
Are the results/ conclusions justified?		4			
How comprehensive and up-to-date is the subject matter presented?		4			
How adequate is the data presentation?		4			
Are units and terminology used correctly?		4			
Is the number of cases adequate?		4			
Are the experimental methods/ clinical studies adequate?					
Is the length appropriate in relation to the content?		4			
Does the reader get new insights from the article?		4			
Please rate the practical significance.		4			
Please rate the accuracy of methods.		4			
Please rate the statistical evaluation and quality control.	5				
Please rate the appropriateness of the figures and tables.	5				
Please rate the appropriateness of the references.	5				
Please evaluate the writing style and use of language.		4			
Please judge the overall scientific quality of the manuscript.		4			
Are the methods used worthy of reproduction in greater deal?		4			

Comments to author: Congratulations on this well written manuscript. I do recommend publication of this study following a minor revision:

1. Page 3, line 9-10. Please rephrase the statement that treatment failure is a frequent event ... ; Glioblastoma recurrence should not be considered as treatment failure. To date , aim of treatment in glioblastoma is not to heal but to delay recurrence while keeping a high health-related quality of life.

2. Page 5 line 3: The letter t in the following is missing
3. Page 6 line 12: please state if these were consecutive cases, if not how many were not analyzed and why ?

Reviewer 2: anonymous

Date received: 27-Jun-2020
Reviewer recommendation: **Return to author for minor modifications**
Reviewer overall scoring: **Medium**

Assessment Form scores: 5 = High/Yes; 3 = Medium/Adequate; 1 = Low

Is the subject area appropriate for the journal		4			
Does the title clearly reflect the paper's content?	5				
Does the abstract clearly reflect the paper's content	5				
Do the keywords clearly reflect the paper's content?	5				
Does the introduction present the problem clearly?	5				
Are the results/ conclusions justified?		4			
How comprehensive and up-to-date is the subject matter presented?		4			
How adequate is the data presentation?		4			
Are units and terminology used correctly?	5				
Is the number of cases adequate?	5				
Are the experimental methods/ clinical studies adequate?		4			
Is the length appropriate in relation to the content?		4			
Does the reader get new insights from the article?		4			
Please rate the practical significance.		4			
Please rate the accuracy of methods.		4			
Please rate the statistical evaluation and quality control.		4			
Please rate the appropriateness of the figures and tables.		4			
Please rate the appropriateness of the references.		4			
Please evaluate the writing style and use of language.		4			
Please judge the overall scientific quality of the manuscript.		4			
Are the methods used worthy of reproduction in greater deal?		4			

Comments to author: The data cover the period 2012-2014. Were the tumours subsequently assigned to the WHO-classification 2016?
It is not specified whether the same surgical techniques were used for all operations: Neuronavigation, 5-ALA, intraoperative MRI and so on
Were the surgical results independent of the surgeon or the experience of the surgeon?
There is no information about postoperative complications, which could also have an influence on the overall result.
No information is given about radiotherapy and chemotherapy or other therapies that could have an influence on overall survival.
Almost one third of patients with SVZ involvement have received only a biopsy and no surgical therapy. What were the reasons for this? Isn't SVZ involvement a phenomenon of tumours that are not operated on for other reasons?

Reviewer 3: anonymous

Date received: 26-May-2020
Reviewer recommendation: **Reject for the following reasons**
Reviewer overall scoring: **Low**

Assessment Form scores: 5 = High/Yes; 3 = Medium/Adequate; 1 = Low

Is the subject area appropriate for the journal					
Does the title clearly reflect the paper's content?			3		
Does the abstract clearly reflect the paper's content	5				
Do the keywords clearly reflect the paper's content?			3		
Does the introduction present the problem clearly?		4			
Are the results/ conclusions justified?		4			
How comprehensive and up-to-date is the subject matter presented?			3		
How adequate is the data presentation?		4			
Are units and terminology used correctly?	5				
Is the number of cases adequate?	5				
Are the experimental methods/ clinical studies adequate?				2	
Is the length appropriate in relation to the content?	5				
Does the reader get new insights from the article?				2	
Please rate the practical significance.				2	
Please rate the accuracy of methods.			3		
Please rate the statistical evaluation and quality control.				2	
Please rate the appropriateness of the figures and tables.				2	
Please rate the appropriateness of the references.		4			
Please evaluate the writing style and use of language.		4			
Please judge the overall scientific quality of the manuscript.			3		
Are the methods used worthy of reproduction in greater deal?		4			

Comments to author: The involvement of the SVZ is highly associated with EOR and surgical morbidity. This result is nothing new. Both EOR and surgical morbidity are proven to be associated with OS. The article does not show that SVZ is an independent prognostic factor. Fig. 2 seems misleading.

Authors' Response to Reviewer Comments

Date received: 22-Jun-2020

Response to reviewer 1

4. Page 3, line 9-10. Please rephrase the statement that treatment failure is a frequent event ... ; Glioblastoma recurrence should not be considered as treatment failure. To date, aim of treatment in glioblastoma is not to heal but to delay recurrence while keeping a high health-related quality of life.

We want to impress our deepest gratitude for the reviewer's response and agree that especially delaying and improving the quality of life must be the main aim for Glioblastom recurrence treatment. This sentence has been changed in the manuscript.

5. Page 5 line 3: The letter t in the following is missing

The letter has jumped into the previous page. Thank you for pointing this out to us.

6. Page 6 line 12: please state if these were consecutive cases, if not how many were not analyzed and why ?

These were indeed all consecutive cases with primary Glioblastoma operated in the time period from 1st January 2012 to 31st December 2014. We have completed this in the result section.

Response to reviewer 2

The data cover the period 2012-2014. Were the tumours subsequently assigned to the WHO-classification 2016?

We want to thank the reviewer for this important remark. Indeed, all glioblastoma cases and molecular properties in our observational database were reviewed with regard to the compliance with the 2016 version of the WHO classification of brain tumors. In this study, we have included the patients with primary glioblastoma multiforme so that cases with secondary and epithelioid glioblastoma, as well as diffuse glioma were not assigned. We inserted this in the Methods section.

It is not specified whether the same surgical techniques were used for all operations: Neuronavigation, 5-ALA, intraoperative MRI and so on

Thank you very much for this essential comment which we have not mentioned in our work. Neuronavigation, the usage of 5-ALA is a standard performance in our department in cases where open surgery was achieved. We wrote this important issue in our methods section for better understanding. Due to the absent of an intraoperative MRI in our department, these scans were not done for glioblastoma surgery. We have also described the stereotactic procedure for completion.

Were the surgical results independent of the surgeon or the experience of the surgeon?

Thanks for this interesting point of view. Operations were performed or accompanied by a senior physician with at least 6 years of experience in neurosurgery. After analyzing our database regarding to this investigation, we could not find a significance distinction between different senior surgeons.

There is no information about postoperative complications, which could also have an influence on the overall result.

We thank the reviewer for his kind and valuable comment. Due to different tumor locations with specific preoperative symptoms, the postoperative complications are heterogenous. These were not statistically significant. After reviewing our data, we have found following complications:

Postoperative Complication of 208 consecutive GB cases	Number of cases	Percentage
Secondary compressive hemorrhage without symptoms	4	1.9 %
Secondary hemorrhage with renewed operation	2	1%
Postoperative Abscess	3	1.4%
Hydrocephalus with Drainage	5	2.4%
Hemianopsia	2	1%
Aphasia	1	0.5%
Hemiparesis	4	1.9%
Meningitis	2	1%
Postoperative cardiopulmonary complication	2	1%
Early postoperative death within 14 days	1	0.5%

No information is given about radiotherapy and chemotherapy or other therapies that could have an influence on overall survival.

Thank you for this important issue. Beside Age, extent of resection and subventricular zone involvement, we have inserted the information about molecular markers like MGMT promoter methylation and IDH1 mutational status as well as physical condition measured by karnofsky performance scale in our multivariate analysis. We assume that these other also well-known parameters like radiotherapy and chemotherapy have their established impact on overall survival and have not proven it in this cohort separately which would be a crucial fact for further investigations.

Almost one third of patients with SVZ involvement have received only a biopsy and no surgical therapy. What were the reasons for this? Isn't SVZ involvement a phenomenon of tumours that are not operated on for other reasons?

Thanks again for your valuable review. We totally agree that the main cases receiving a stereotactic biopsy showed a significant incidence of SVZ involvement (38/46, 82.6%). They were also associated with higher rate of multifocal growth pattern (63/77, 81.8%) which is in our opinion the main reason for a decision of biopsy due to the failed high resection in the already surgical planning. This important comment has been implemented in the discussion section.

Response to reviewer 3

The involvement of the SVZ is highly associated with EOR and surgical morbidity. This result is nothing new. Both EOR and surgical morbidity are proven to be associated with OS. The article does not show that SVZ is an independent prognostic factor. Fig. 2 seems misleading.

We want to thank the reviewer for his opinion and comment. In the multivariate analysis, we could show that SVZ involvement has an independent influence on overall survival. An accurate examination of figure 2 was performed after review.

Reviewers' Comments to Revised Submission

Reviewer 1: anonymous

Date received: 07-Jul-2020
Reviewer recommendation: **Accept in present form**
Reviewer overall scoring: **High**

Assessment Form scores: 5 = High/Yes; 3 = Medium/Adequate; 1 = Low

Is the subject area appropriate for the journal		4			
Does the title clearly reflect the paper's content?		4			
Does the abstract clearly reflect the paper's content		4			
Do the keywords clearly reflect the paper's content?		4			
Does the introduction present the problem clearly?		4			
Are the results/ conclusions justified?		4			
How comprehensive and up-to-date is the subject matter presented?		4			
How adequate is the data presentation?		4			
Are units and terminology used correctly?		4			

Is the number of cases adequate?		4			
Are the experimental methods/ clinical studies adequate?					
Is the length appropriate in relation to the content?		4			
Does the reader get new insights from the article?		4			
Please rate the practical significance.		4			
Please rate the accuracy of methods.			3		
Please rate the statistical evaluation and quality control.		4			
Please rate the appropriateness of the figures and tables.		4			
Please rate the appropriateness of the references.		4			
Please evaluate the writing style and use of language.			3		
Please judge the overall scientific quality of the manuscript.		4			
Are the methods used worthy of reproduction in greater deal?		4			

Comments to author: Well done!

Reviewer 2: Anonymous

Date received: 27-Jun-2020
Reviewer recommendation: **Accept in present form**
Reviewer overall scoring: **Medium**

Assessment Form scores: 5 = High/Yes; 3 = Medium/Adequate; 1 = Low

Is the subject area appropriate for the journal		4			
Does the title clearly reflect the paper's content?	5				
Does the abstract clearly reflect the paper's content	5				
Do the keywords clearly reflect the paper's content?	5				
Does the introduction present the problem clearly?	5				
Are the results/ conclusions justified?		4			
How comprehensive and up-to-date is the subject matter presented?		4			
How adequate is the data presentation?		4			
Are units and terminology used correctly?	5				
Is the number of cases adequate?	5				
Are the experimental methods/ clinical studies adequate?		4			
Is the length appropriate in relation to the content?		4			
Does the reader get new insights from the article?	5				
Please rate the practical significance.		4			
Please rate the accuracy of methods.		4			
Please rate the statistical evaluation and quality control.		4			
Please rate the appropriateness of the figures and tables.	5				
Please rate the appropriateness of the references.	5				
Please evaluate the writing style and use of language.	5				
Please judge the overall scientific quality of the manuscript.		4			
Are the methods used worthy of reproduction in greater deal?		4			

Comments to author: Thank you very much for the implementation of the comments. I recommend the publication of this well-written manuscript.
